# N-acetylcysteine attenuates myocardial dysfunction and postischemic injury by restoring caveolin-3/eNOS signaling in diabetic rats

**DOI:** 10.1186/s12933-016-0460-z

**Published:** 2016-10-12

**Authors:** Wating Su, Yuan Zhang, Qiongxia Zhang, Jinjin Xu, Liying Zhan, Qiqi Zhu, Qingquan Lian, Huimin Liu, Zhong-yuan Xia, Zhengyuan Xia, Shaoqing Lei

**Affiliations:** 1 Department of Anesthesiology, Renmin Hospital of Wuhan University, Wuhan, China; 2 Department of Anesthesiology, The Second Affiliated Hospital & Yuying Children’s Hospital of Wenzhou Medical University, Wenzhou, Zhejiang China; 3 Department of Anesthesiology, The University of Hong Kong, Hong Kong SAR, China

**Keywords:** N-acetylcysteine, Diabetic cardiomyopathy, Myocardial ischemia–reperfusion injury, Caveolin-3, Diabetes

## Abstract

**Background:**

Patients with diabetes are prone to develop cardiac hypertrophy and more susceptible to myocardial ischemia–reperfusion (I/R) injury, which are concomitant with hyperglycemia-induced oxidative stress and impaired endothelial nitric oxide (NO) synthase (eNOS)/NO signaling. Caveolae are critical in the transduction of eNOS/NO signaling in cardiovascular system. Caveolin (Cav)-3, the cardiomyocytes-specific caveolae structural protein, is decreased in the diabetic heart in which production of reactive oxygen species are increased. We hypothesized that treatment with antioxidant N-acetylcysteine (NAC) could enhance cardiac Cav-3 expression and attenuate caveolae dysfunction and the accompanying eNOS/NO signaling abnormalities in diabetes.

**Methods:**

Control or streptozotocin-induced diabetic rats were either untreated or treated with NAC (1.5 g/kg/day, NAC) by oral gavage for 4 weeks. Rats in subgroup were randomly assigned to receive 30 min of left anterior descending artery ligation followed by 2 h of reperfusion. Isolated rat cardiomyocytes or H9C2 cells were exposed to low glucose (LG, 5.5 mmol/L) or high glucose (HG, 25 mmol/L) for 36 h before being subjected to 4 h of hypoxia followed by 4 h of reoxygenation (H/R).

**Results:**

NAC treatment ameliorated myocardial dysfunction and cardiac hypertrophy, and attenuated myocardial I/R injury and post-ischemic cardiac dysfunction in diabetic rats. NAC attenuated the reductions of NO, Cav-3 and phosphorylated eNOS and mitigated the augmentation of O_2_^−^, nitrotyrosine and 15-F2t-isoprostane in diabetic myocardium. Immunofluorescence analysis demonstrated the colocalization of Cav-3 and eNOS in isolated cardiomyocytes. Immunoprecipitation analysis revealed that diabetic conditions decreased the association of Cav-3 and eNOS in isolated cardiomyocytes, which was enhanced by treatment with NAC. Disruption of caveolae by methyl-β-cyclodextrin or Cav-3 siRNA transfection reduced eNOS phosphorylation. NAC treatment attenuated the reductions of Cav-3 expression and eNOS phosphorylation in HG-treated cardiomyocytes or H9C2 cells. NAC treatment attenuated HG and H/R induced cell injury, which was abolished during concomitant treatment with Cav-3 siRNA or eNOS siRNA.

**Conclusions:**

Hyperglycemia-induced inhibition of eNOS activity might be consequences of caveolae dysfunction and reduced Cav-3 expression. Antioxidant NAC attenuated myocardial dysfunction and myocardial I/R injury by improving Cav-3/eNOS signaling.

## Background

Cardiovascular disease is a leading cause of morbidity and mortality especially in patients with diabetes mellitus (DM) [1]. Patients with DM are prone to develop multiple cardiovascular complications, including coronary heart disease, cardiac hypertrophy and heart failure [2]. Most diabetic heart failure etiology concerns ischemic heart diseases [e.g., myocardial ischemia/reperfusion (I/R) injury] and diabetic cardiomyopathy [3, 4]. The pathogenesis of diabetic cardiomyopathy and myocardial I/R injury is very complicated, but much evidence indicates the involvement of excessive production of reactive oxygen species (ROS) induced by metabolic disorders in diabetes [2, 5, 6]. Despite significant advances in laboratory researches and clinical trials of antioxidant treatment in the past decade, the underlying mechanisms by which hyperglycemia-induced oxidative stress exerts adverse effects in diabetic hearts are not yet fully understood.

Nitric oxide (NO), which is synthesized by a family of NO synthases (NOS) including neuronal, inducible, and endothelial NOS (n/i/eNOS), plays an important role in cardiovascular physiology and pathology [7]. The eNOS-derived NO has been reported to inhibit the progression of myocardial infarction [8], ameliorate myocardial I/R injury [9] and left ventricular hypertrophy [10, 11], and prevent the onset of heart failure [12]. Moreover, NO can scavenge ROS and reduce detrimental effects of ROS [13, 14]. Therefore, regulation of the eNOS/NO and ROS balance is of importance in the progression of diabetic cardiomyopathy and myocardial I/R injury in diabetes. eNOS is constitutively expressed in the heart and enriched in cardiomyocyte caveolae [15, 16]. Caveolae serves as a platform in plasma membrane to modulate transduction pathways via signaling molecules docked within caveolins, and three integral isoforms of caveolins are identified in mammalian caveolae, termed caveolin (Cav) 1, 2 and 3. In the cardiovascular system, Cav-1 and Cav-2 are found in multiple cell types, whereas Cav-3 is mainly expressed in cardiac muscle cells and is essential for the formation of cardiomyocytes caveolae [17]. In cardiomyocytes, eNOS localizes to caveolae bound to Cav-3, and the co-localization of Cav-3 and eNOS may facilitate both eNOS activation and NO release for intercellular signaling [18]. Therefore, Cav-3 is important for maintaining eNOS/NO signaling in the heart. Thus, any alteration of Cav-3 expression in diabetic condition may be implicated in the pathogenesis of diabetic cardiomyopathy and myocardial I/R injury. This notion is supported by our previous findings that decreased Cav-3 expression and cardiac NO bioavailability are detected in hearts from rats with chronic streptozotocin (STZ)-induced diabetes [19, 20], which are associated with more serious myocardial I/R injury [19, 21]. However, it remains unclear whether or not excessive production of ROS mediated diabetic abnormalities is an independent manifestation of hyperglycemic injury or is linked to impaired Cav-3 expression and eNOS/NO signaling in diabetes. In the present study, we hypothesize hyperglycemia-induced oxidative stress promotes caveolae dysfunction and impairs Cav-3/eNOS signaling. Our data suggested that inhibition of excessive oxidative stress by N-acetylcysteine (NAC), an antioxidant which has been proven by us [22–26] and others [27, 28] to be effective in attenuating oxidative stress in diabetic hearts, could restore Cav-3 expression and improve eNOS/NO signaling, which ultimately attenuate diabetic cardiomyopathy and myocardial I/R injury in diabetic rats.

## Methods

### Experimental animals and induction of diabetes

This study was carried out in accordance with the recommendations in the Guide for the Care and Use of Laboratory Animals of the National Institutes of Health (NIH Publication No. 80–23), and approved by Institutional Animal Care and Use Committee of Wuhan University. Male Sprague-Dwaley rats (260 ± 10 g, 7–8 weeks) were obtained from the Laboratory Animal Unit of Wuhan University. They were randomly assigned to the control group, diabetic group, and diabetic group treated with NAC. Diabetes was induced by a single tail vein injection of streptozotocin (STZ, Sigma, USA) at the dose of 60 mg/kg under halothane anesthesia, while control rats were injected with equal volume citrate buffer alone. Three days after STZ injection, the rats with fasting plasma glucose levels ≥16.7 mM measured by using a glucose analyzer (Beckman Instruments), were considered as diabetes and used for the experiments. One week after induction of diabetes, rats were treated by oral gavage with vehicle or NAC for 4 weeks at the dose of 1.5 g/kg/day [23]. Rats in subgroups were proceeded to evaluate cardiac function by echocardiography. After measurement of plasma glucose and body weight, the rats were euthanized under deep anesthesia and the hearts were rapidly harvested and stored at −80 °C for further detection.

### Cardiac function by echocardiography

Transthoracic echocardiography was performed noninvasively at experiment termination with Vevo 770™ high-resolution imaging system equipped with a 17.0 MHz transducer (RMV-716, Visual Sonics, Canada), and left ventricular (LV) dimensions and LV diastolic and systolic function were assessed by M-mode and Doppler echocardiography as we previously described [20]. The heart rate (HR), left ventricular internal dimensions at end systole (LVIDs) and diastole (LVIDd), left ventricular posterior wall dimensions at end diastole (LVPWd) and systole (LVPWs), LV end-distolic volume (LVVd) and end-systolic volume (LVVs), left ventricular isovolumic relaxation time (IVRT), stroke volume (SV) and the ratio of the peak velocity of early (E) and late (A) diastolic filling (E/A) were monitored. Fractional shorting (FS,  %) = (LVIDd − LVIDs)/LVIDd ×100 %, LVPW (%) = (LVPWs − LVPWd)/LVIDd ×100 %, Ejection fraction (EF,  %) = (LVVd − LVVs)/LVVd ×100 %. All derived measures by echocardiography were obtained by averaging the readings of three consecutive beats.

### Haematoxylin and eosin (H-E) staining and in situ DHE (dihydroethidium) staining of the left ventricle

Cardiomyocytes cross-sectional area was assessed by H-E stained paraffin-embedded sections of left ventricles (LV), and in situ levels of LV O_2_^−^ production were determined by DHE staining, as we previously described [24]. A minimum of 150 cells per rat was chosen for analysis of cardiomyocytes cross-sectional area. The fluorescence of DHE-stained positive cells was calculated in each of five randomly selected fields.

### Rat cardiomyocytes isolation

Adult rat cardiomyocytes were isolated and prepared via a modified method, as described [20]. Briefly, the rats were anesthetized and heparinized, and the hearts were rapidly removed and mounted on a Langendorff perfusion apparatus. After enzymatic digestion for 20 min (min), the ventricle was gently teased into small pieces and filtered immediately to exclude undigested tissues, and then the cardiomyocytes were pelleted by gravity for 10 min and resuspended with M199 medium for three times. After the final suspension, the cardiomyocytes were incubated in low glucose (LG) (5.5 mmol/L), high glucose (HG) (25 mmol/L), mannitol/glucose (19.5 mmol/L mannitol plus 5.5 mmol/L glucose) or N-acetylcysteine (NAC, 1 mmol/L [26]) at 37 °C in Medium 199 (Gibco, Grand Island, NY) medium with selenium/insulin/transferring (Sigma-Aldrich, St. Louis, MO) containing various treatments for 36 h (h) according to the previous study [20], in which high glucose duration for 36 h significantly induced cardiomyocytes injury and impaired Cav-3 expression.

### Confocal immunofluorescence

The subcellular localization of Cav-3 and eNOS in cardiomyocytes was inspected by confocal immunofluorescense, as described [20]. Briefly, isolated cardiomyocytes were plated on Matrigel precoated glass coverslips, incubated in Medium 199 for 36 h (h). The cells were fixed and blocked with 10 % goat serum and 1 % BSA, and then incubated with a mixture of rabbit against rat eNOS antibody (1:100; Santa Cruz Biotechnology) and mouse against rat Cav-3 antibody (1:50; Santa Cruz Biotechnology, Santa Cruz, CA, USA). Then, the cells were incubated for a mixture of Alexa Fluor 488 goat anti-mouse IgG and Alexa Fluor 594 goat anti-rabbit IgG (1:2000; Invitrogen, Carlsbad, CA, USA), and prepared for confocal laser scanning microscopic imaging with mounting medium containing DAPI (Vector Laboratories, Burlingame, CA, USA).

### Immunoprecipitation

Immunoprecipitation was performed as previously described [20]. Briefly, the isolated cardiomyocytes were cultured in LG or HG conditions with or without NAC treatment for 36 h. The cells were homogenized in lysis buffer and the protein concentration was measured by Bio-Rad Protein Assay kit. A total of 500 mg cell preparations were performed to immunoprecipitation with 2 mg Cav-3 primary antibody in the presence of 20 mL protein A/G plus-agarose. Then, the immunoprecipitates were denatured and subjected to analysis for eNOS expression by Western blot as described below.

### Myocardial I/R injury model

After the completion of NAC treatment (5-weeks after the onset of diabetes), subgroup of the experimental rats was subjected to myocardial I/R injury as described previously [21, 29], in which rats were anaesthetized and subjected to myocardial I/R achieved by occluding the left anterior descending coronary artery for 30 min followed by reperfusion for 2 h. Sham-operated rats underwent the same surgical procedures without ligation. At the end of the reperfusion, myocardial infarct size of the experimental rats was measured using Evans Blue dye/2,3,5-triphenyltetrazolium chloride (TTC) staining as we described [29]. The unstained region by Evans blue dye was considered as the area at risk (AAR). The area unstained by TTC was identified as the infarcted tissue. Myocardial infarct size was expressed as a percentage of the AAR (%AAR). Hemodynamic changes of the experimental rats subjected to myocardial I/R were monitored as we described [29]. Briefly, a saline-filled catheter was inserted into the left ventricle via an incision on the right common carotid artery and then connected to a pressure transducer. The left ventricular systolic pressure (LVSP), left ventricular maximum rate of increase of left ventricular developed pressure (+dp/dt), maximum rate of decrease of left ventricular developed pressure (−dp/dt) and HR were monitored at 10 min before ischemia (baseline) and 2 h after reperfusion.

### siRNA studies in H9C2 cells and induction of hypoxia/reoxygenation

Embryonic rat cardiomyocyte-derived cell line H9C2 was maintained in DMEM (Dulbecco’s modified Eagle’s medium) containing 10 % FBS in a humidified atmosphere (5 % CO_2_) at 37 °C. Commercial Cav-3 siRNA or eNOS siRNA (Santa Cruz Biotechnology) was used for inhibition of Cav-3 expression according to the manufacturer’s protocol. After transfection with control, Cav-3 siRNA or eNOS siRNA, cells were treated with N-acetylcysteine (NAC, 1 mmol/L) in the condition of LG or HG for 36 h, then the cells was exposed to 4 h of hypoxia (1 % O_2_, 5 % CO_2_, 94 % N_2_) followed by 4 h of reoxygenation (H/R). LDH (lactate dehydrogenase) release was measured to evaluate the extent of cellular injury using a cytotoxicity assay kit (Jiancheng Bioengineering Institute, China) according to the manufacturer’s instructions.

### Determination of CK-MB and 15-F2t-isoprostane

Serum CK-MB (creatine kinase-MB) levels, a major indicator of myocardial I/R injury, were measured in the arterial blood samples collected at the end of reperfusion by enzyme immunoassay using a commercial ELISA kit (Elabscience Biotechnology) as we described [29]. 15-F2t-isoprostane (15-F2t-IsoP), a specific marker of oxidative stress [30], was measured using an EIA kit (Cayman chemical, Ann Arbor, MI, USA) as described previously [23]. The value of 15-F2t-IsoP in the heart tissue was expressed as pg/mg protein.

### Measurement of the levels of O_2_^−^, NO and nitrotyrosine

O_2_^−^ production in cardiac tissues or isolated cardiomyocytes was assayed using lucigenin chemiluminescence method [31, 32]. Total protein concentration in the supernatant was determined by Bio-Rad Protein Assay kit. The supernatant samples were loaded with dark-adapted lucigenin (5 μM), then read in 96-well microplates using a luminometer (GloMax, Promega) with and without pretreatment with the NOS inhibitor L-NAME (100 μM [33]) for 30 min at room temperature. Light emission was recorded for 5 min and expressed as mean light units (MLU)/min/100 μg protein. NO content was determined using Lowry assay kit (Bio-Rad, CA, USA). The concentration of nitrites (NO_2_^−^) and nitrates (NO_3_^−^), stable end products of nitric oxide (NO), were determined by the Griess reaction, as described previously [34] NO levels were expressed as nmol/μg protein. The levels of myocardial nitrotyrosine in the collected supernatant were assayed by chemiluminescence detection using the Nitrotyrosine Assay Kit according to the manufacturer’s instructions (Millipore, USA). Myocardial nitrotyrosine concentration was expressed as μg/mg protein.

### Western blot analysis

Equal amounts of protein were separated via 7.5–12.5 % SDS-PAGE and subsequently transferred to PVDF membrane for immunoblot analysis as described previously [23]. The membranes were blocked in 5 % no fat milk for 2 h at room temperature, and then incubated overnight at 4 °C with primary antibodies against Cav-3 (1:500, Santa Cruz Biotechnology), eNOS (1:1000, Cell Signaling Technology), p-eNOS (Ser1177) (1:500, Cell Signaling Technology). After being washed with TBST, the membranes were incubated with proper secondary horseradish peroxidase (HRP)-conjugated antibodies (1:5000–1:10,000, Cell Signaling Technology), and developed with enhanced chemiluminescence reagent (GE Healthcare, USA). The membranes were subsequently reblotted for GAPDH (1:2000, Cell Signaling Technology), and the results were normalized to GAPDH to correct for loading. Data are presented as percent change relative to the measurement in control rats.

### Statistical analysis

All the values are expressed as mean ± SD GraphPad Prism software program (GraphPad Software Inc., San Diego, CA, USA) was used for statistical analysis. Comparison between multiple groups was made with one-way analysis of variance (ANOVA) followed by Tukey’s test for multiple comparisons, or two-way repeated-measures ANOVA followed by Bonferroni’s post hoc test in grouped values. Significance was defined as P < 0.05.

## Results

### Antioxidant NAC attenuated cardiac hypertrophy and myocardial dysfunction in diabetic rats

At the end of treatment period, the diabetic rats without NAC treatment had significantly elevated plasma glucose and reduced heart weight compared with control rats, which was not altered by 4 weeks of treatment with NAC. However, the decreased body weight and increased ratio of heart weight to body weight in diabetic rats, an indirect index of myocardial hypertrophy, was significantly reversed by NAC treatment (Table 1). We further assessed the cardiomyocyte cross-sectional area by H-E staining in LV sections. The cross-sectional area in untreated diabetic rats (452.5 ± 63.7 μm^2^) was significantly bigger than that in control rats (301.5 ± 41.2 μm^2^), NAC treatment (338.8 ± 47.1 μm^2^) significantly attenuated this alteration of cross-sectional area in diabetic rats, indicating antioxidant treatment with NAC can attenuate cardiomyocyte hypertrophy in diabetes.

**Table 1:** General characteristics of experimental group

	C	D	D + NAC
Plasma glucose (mM)	6.41 ± 0.61	29.12 ± 5.13**	25.86 ± 2.76**
Heart weight (g)	1.62 ± 0.23	1.21 ± 0.17*	1.38 ± 0.20*
Body weight (g)	450.5 ± 20.3	263.7 ± 17.6*	362.5 ± 24.2*^#^
Heart weight/bodyweight (mg/g)	3.59 ± 0.09	4.58 ± 0.12*	3.81 ± 0.11^#^

Control (*C*) or STZ-induced diabetic (*D*) rats were either untreated or treated with N-acetylcysteine (1.5 g/kg/day, D + NAC) by oral gavage for 4 weeks. All the results are expressed as mean ± SD, n = 8. Differences in general characteristics among the three groups were determined by using one-way ANOVA followed by Tukey’s test

* P < 0.05

** P < 0.01 vs. C group

^#^ *P* < 0.05 vs. D group

**Fig. 1:**
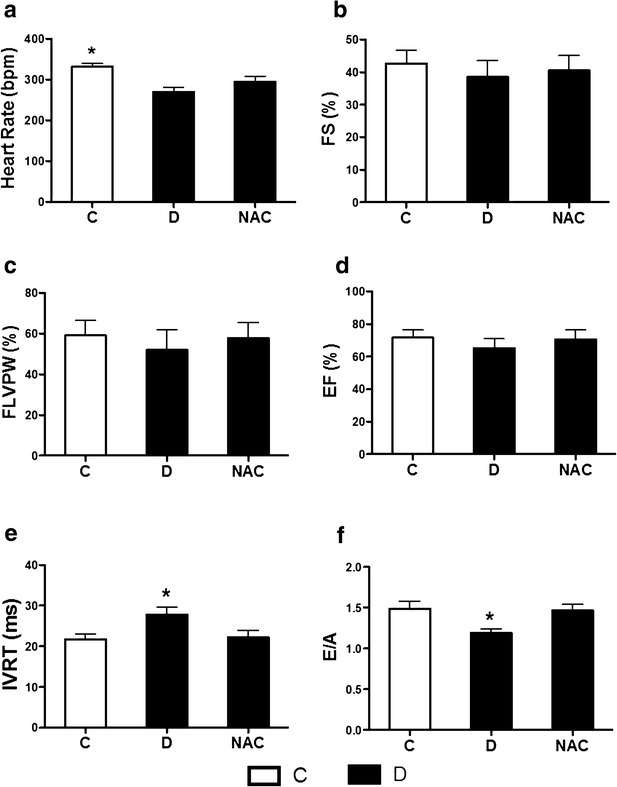
N-acetylcysteine treatment attenuated myocardial dysfunction in diabetic rats. Control (*C*) or STZ-induced diabetic rats were either untreated (*D*) or treated with antioxidant N-acetylcysteine (1.5 g/kg/day, NAC) by oral gavage for 4 weeks. All the rats were lightly anesthetized and performed transthoracic echocardiography. **a**–**f** Heart rate (**a**), fractional shortening (FS) (**b**), fractional left ventricular posterior wall thickening (FLVPW) (**c**), ejection fraction (EF) (**d**), left ventricular isovolumic relaxation time (IVRT) (**e**), and the ratio of peak velocity of early and late diastolic filling (E/A) (**f**). All the results are expressed as mean ± SD, n = 7 per group. Differences were determined by using one-way ANOVA followed by Tukey’s test. *P < 0.05 vs. all the other groups

We further examined the effects of NAC on cardiac function by echocardiography. Parameters in FS (Fig. 1b), FLVPW (Fig. 1c) and EF (Fig. 1d) did not significantly differ among all the experimental groups. This means that the systolic function was not yet affected in 5 weeks of diabetic rats. Further, diabetic rats induced by STZ for 5 weeks manifested decreased heart rate (HR) (Fig. 1a) and reduced ratio of peak velocity of early and late diastolic filling (E/A) (Fig. 1f), accompanied with increased left ventricular isovolumic relaxation time (IVRT) (Fig. 1e), which indicated diastolic function in early diabetic heart was impaired, and this is consistent with the characteristics of diabetic cardiomyopathy [35–37]. NAC treatment for 4 weeks restored the levels of E/A and IVRT, but did not significantly increase HR (Fig. 1a, e and f).

### Effects of antioxidant NAC on the levels of 15-F2t-IsoP, NO, nitrotyrosine and O_2_^−^ in diabetic heart tissues

**Fig. 2:**
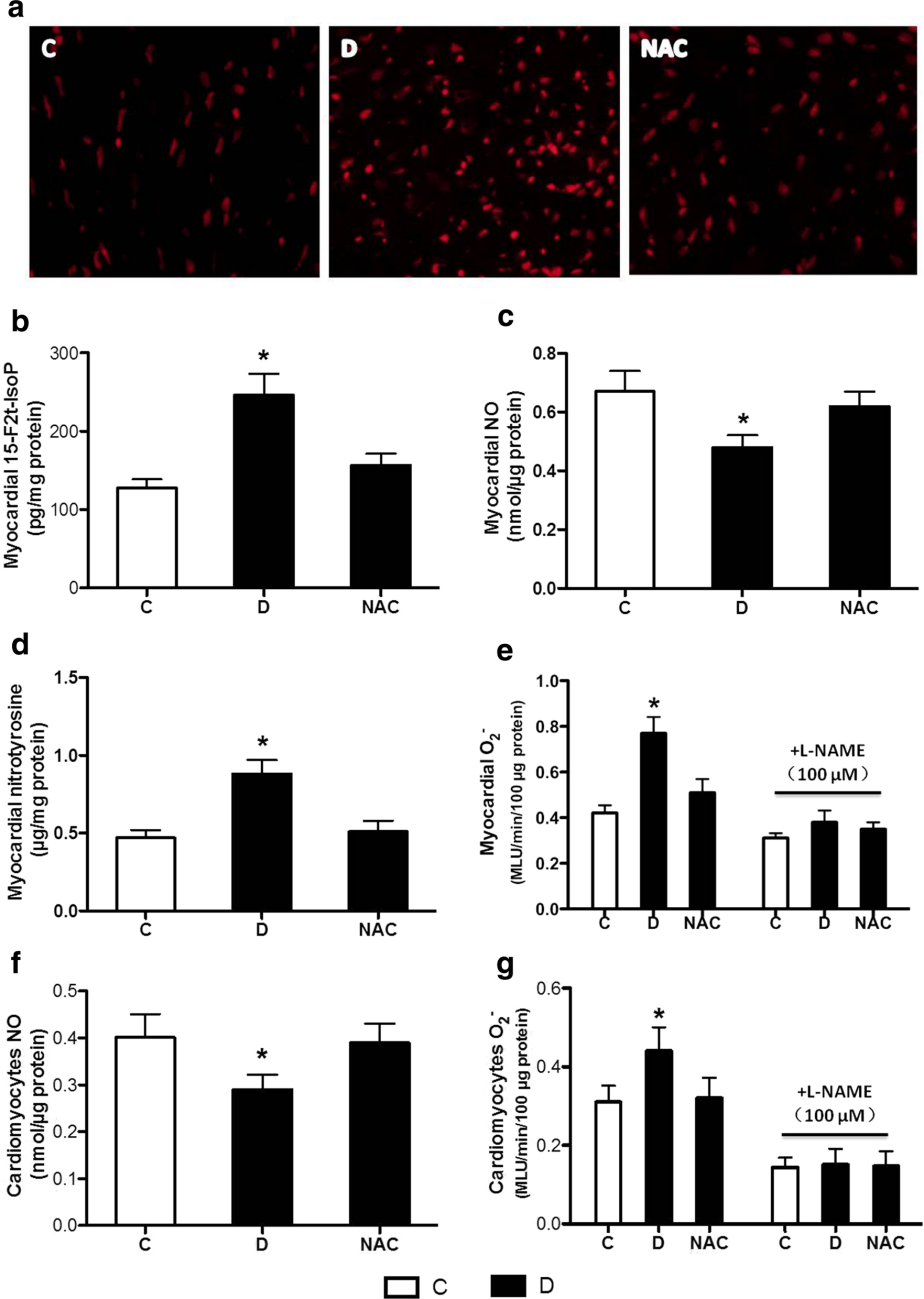
Effects of N-acetylcysteine treatment on the levels of 15-F2t-IsoP, NO, nitrotyrosine and O_2_^−^ in diabetic myocardium, as well as NO and O_2_^−^ levels in isolated cardiomyocytes. Control (*C*) or STZ-induced diabetic rats were either untreated (*D*) or treated with antioxidant N-acetylcysteine (1.5 g/kg/day, NAC) by oral gavage for 4 weeks,and adult rats cardiomyocytes were isolated and prepared. **a**–**e** In situ O_2_^−^ production in LV sections stained by DHE (**a**), myocardial levels of 15-F2t-IsoP (**b**), NO (**c**), nitrotyrosine (**d**) and O_2_^−^ in the absence and presence of L-NAME (**e**); **f**, **g** cardiomyocytes levels of NO (**f**) and O_2_^−^ (**g**) production in the absence and presence of L-NAME. All the results are expressed as mean ± SD, n = 7. Differences of O_2_^−^ in the absence and presence of L-NAME were determined by using two-way repeated-measures ANOVA followed by Bonferroni’s post hoc test, the others were determined by using one-way ANOVA followed by Tukey’s test. *P < 0.05 vs. all the other groups. MLU, mean light unit

Oxidative stress plays an important role in the pathogenesis of diabetic complication. In the present study, we detected myocardial O_2_^−^ production in LV sections stained by DHE. The number of DHE-stained positive cells in the hearts of diabetic rats was much more than that in control rats, which was attenuated by NAC treatment (Fig. 2a**)**. We then measured the levels of 15-F2t-IsoP, a specific marker of oxidative stress. As shown in Fig. 2b, the level of 15-F2t-IsoP in diabetic group was significantly higher than that of the control group, which was attenuated by NAC treatment.

We also detected the levels of NO, nitrotyrosine and O_2_^−^ production in homogenized heart tissues. As is shown in Fig. 2c–e, diabetes significantly decreased NO levels, and increased nitrotyrosine and O_2_^−^ production in heart tissues. NAC treatment for 4 weeks attenuated or blocked all these alterations. Further studies revealed that diabetes induced increase of O_2_^−^ levels was blocked by the NOS inhibitor L-NAME (100 μM) (Fig. 2e), indicating a NOS-dependent mechanism for O_2_^−^ accumulation or “eNOS uncoupling”. To confirm this, we also detected the levels of NO and O_2_^−^ production in isolated cardiomyocytes. As shown in Fig. 2f, g, the levels of NO were significantly decreased, and O_2_^−^ production was significantly increased in isolated cardiomyocytes from diabetic rats as compared with that from control rats, NAC treatment reversed these changes. Similar to the treatment effects of NOS inhibitor L-NAME in homogenized heart tissues, the increased O_2_^−^ production was also blocked by L-NAME in isolated cardiomyocytes.

### Effects of NAC treatment on myocardial expression of Cav-3 and p-eNOS in diabetic rats, as well as their association in isolated cardiomyocytes

**Fig. 3:**
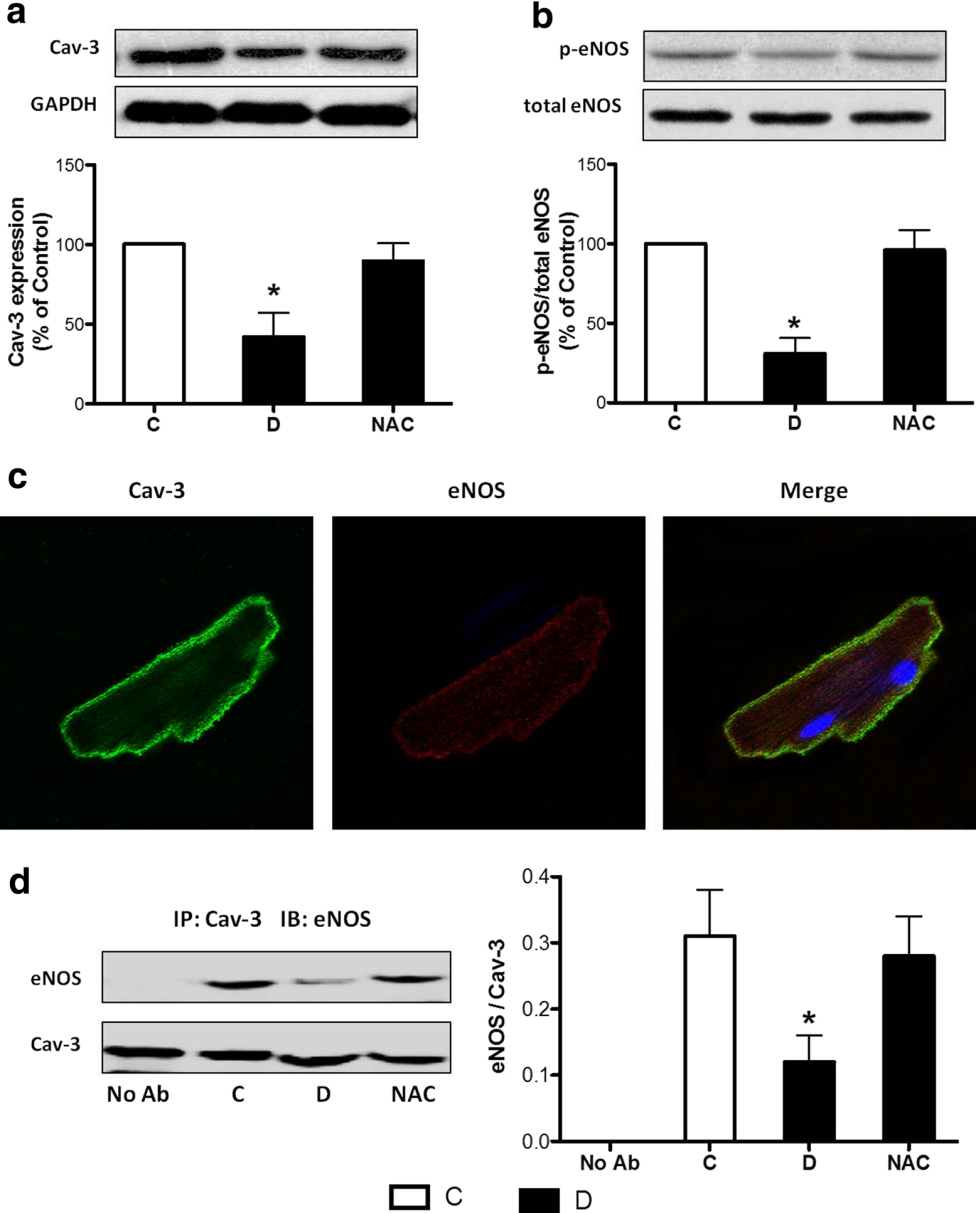
Expression of Cav-3 and p-eNOS in diabetic hearts, and their association in isolated cardiomyocytes. **a**, **b** Representative western blot of Cav-3 expression with glyceraldehyde-3-phosphate dehydrogenase (GAPDH) as loading control (**a**) and p-eNOS expression compared with total eNOS expression (**b**) in myocardium from the control (**c**), diabetic (**d**) and N-acetylcysteine-treated (1.5 g/kg/day, NAC) rats. **c** Confocal laser microscopic image of isolated cardiomyocytes from non-diabetic rats underwent standard immunofluorescence staining with Cav-3 and eNOS. **d** The lysates of isolated rat cardiomyocytes from various groups containing equal amount of total protein were subjected to immunoprecipitation (IP) with Cav-3 antibody (Ab) and analyzed by immunoblot (IB) with eNOS and Cav-3 antibody. All the results are expressed as mean ± SD, n = 7. Differences were determined by using one-way ANOVA followed by Tukey’s test. *P < 0.05 vs. all the other groups

We previously reported decreased Cav-3 expression (a muscle-specific marker of caveolae [17]) and reduced phosphorylation of eNOS in the hearts of diabetic rats [20], which may be attributable to reduced tolerance of diabetic heart to myocardial I/R [7, 21]. Therefore, our present study examined whether NAC treatment could reverse these changes in the diabetic hearts. As shown in Fig. 3a, b, NAC significantly attenuated the reductions of cardiac Cav-3 and p-eNOS expression in diabetic rats. We next examined the association of Cav-3 and eNOS in isolated cardiomyocytes. As shown in Fig. 3c, the confocal immunofluorescence staining showed the colocalization between eNOS and Cav-3 during basal conditions in the cell membrane (indicated by scant yellow punctate staining of the cell periphery) of insolated cardiomyocytes from non-diabetic rats, which may facilitate the transduction of eNOS/NO signaling [18]. To confirm this, we also did immunoprecipitation experiments in isolated rat cardiomyocytes from various groups. A mount of eNOS remained constitutively associated with Cav-3 during basal conditions, while the diabetic conditions decreased the association of eNOS with Cav-3, which was restored by NAC treatment (Fig. 3d).

### Hyperglycemia-induced inhibition of eNOS activation involves caveolae dysfunction and reduced Cav-3 expression, which contributes to eNOS uncoupling

**Fig. 4:**
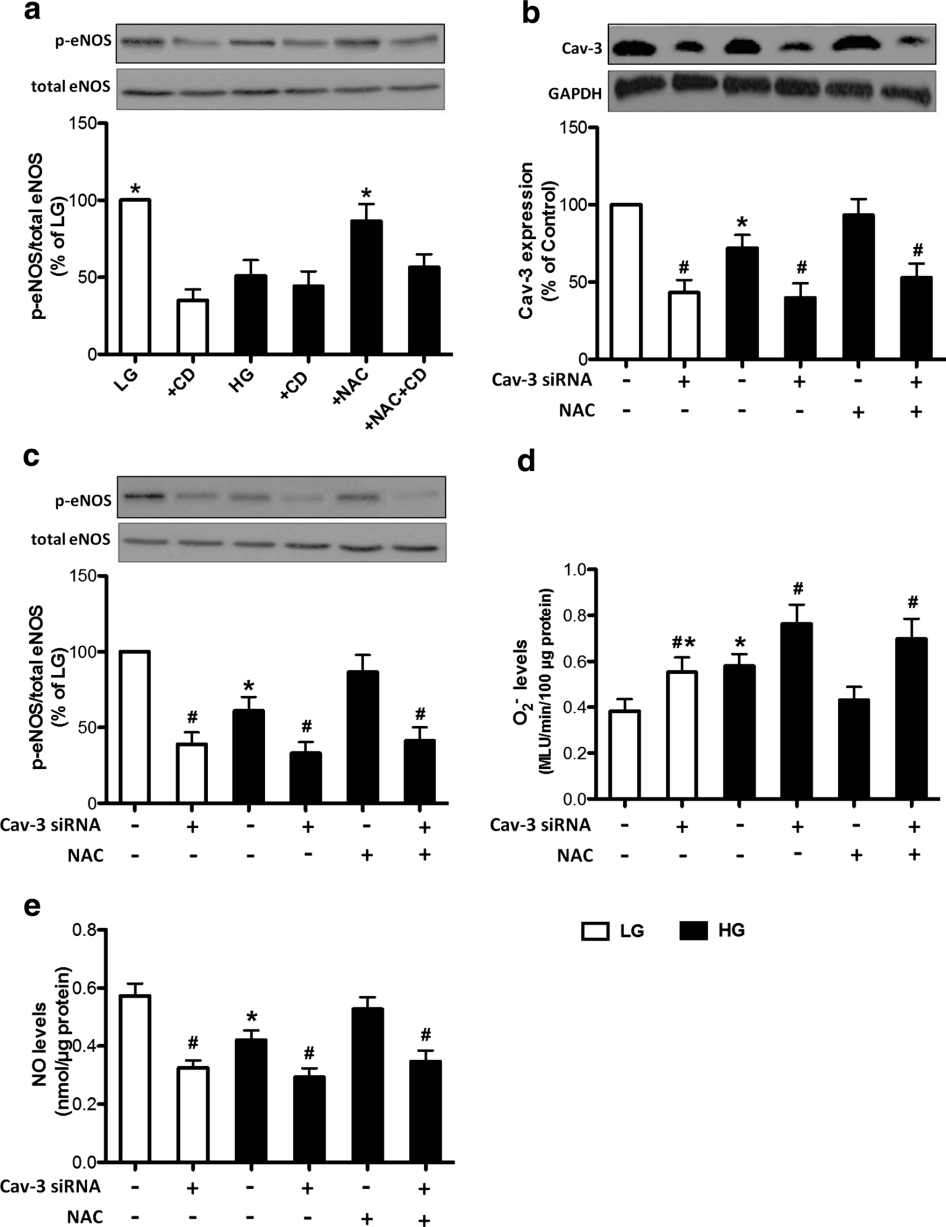
Expression of p-eNOS and Cav-3 in cultured cardiomyocytes and H9C2 cells after various treatment in LG (5.5 mmol/L) and HG (25 mmol/L) conditions. **a** Representative western blot of p-eNOS (Ser^1177^) in comparison with total eNOS expression in cardiomyoytes exposed to HG in the presence of N-acetylcysteine (NAC, 1 mmol/L) or methyl-b-cyclodextrin (CD, 10 μmol/L); **b**–**e** Representative western blot of Cav-3 (**b**) and p-eNOS (Ser^1177^) (**c**), and levels of O_2_^−^ (**d**) and NO (**e**) in H9C2 cells transfected with Cav-3 siRNA or treated with NAC (1 mmol/L). All the results are expressed as mean ± SD, n = 7. Differences were determined by using one-way ANOVA followed by Tukey’s test. ^#^P < 0.05 vs. corresponding group without siRNA treatment, *P < 0.05 vs. all the other groups

Given cardiomyocytes caveolae is necessary for eNOS signaling [18], we determined whether caveolae dysfunction is crucial in hyperglycemia-induced inhibition of eNOS activation. In our preliminary studies, the cav-3 expression and eNOS phosphorylation at ser^1177^ were not significantly affected by NAC or mannitol in isolated cardiomyocytes or H9C2 cells (data not shown). As shown in Fig. 4a, in the presence of methyl-b-cyclodextrin (CD, 10 μmol/L), a disrupter of cholesterol-rich caveolae [38], the phosphorylation of eNOS was reduced significantly in cardiomocytes during LG conditions, suggesting that eNOS activation required caveolae under basal conditions. Disruption of caveolae by CD slightly but did not further significantly exaggerate the reduction of eNOS phosphorylation in HG-treated cardiomyocytes. NAC treatment restored eNOS phosphorylation in HG-treated cardiomyocytes, but this effect was abolished during concomitant CD treatment. We next investigated whether hyperglycemia-induced inhibition of eNOS activation might be associated with reduced cav-3 expression. As shown in Fig. 4b, H9C2 cells treated with rat-specific Cav-3 siRNA exhibited about 57 % reduction of Cav-3 expression during both LG and HG conditions. This reduction of Cav-3 expression resulted in further reduced eNOS phosphorylation in both LG- and HG-treated cells (Fig. 4c). NAC treatment restored Cav-3 expression and eNOS phosphorylation in HG-treated cells, which was abolished during concomitant treatment with Cav-3 siRNA (Fig. 4b, c). Therefore, the beneficial effects of NAC on eNOS phosphorylation in HG-treated cells require normal caveolae and Cav-3 expression.

We then examined the treatment effects of Cav-3 siRNA on the levels of O_2_^−^ and NO in HG-treated H9C2 cells. As shown in Fig. 4d, e, HG significantly increased the levels of O_2_^−^ and decreased the levels of NO compared to LG. Cav-3 siRNA further increased these changes of O_2_^−^ and NO. NAC treatment significantly attenuated HG-induced increase of O_2_^−^ production and decrease of NO levels. While these beneficial effects of NAC were abolished by transfection of Cav-3 siRNA. These results collectively suggest that reduced Cav-3 expression contributes to eNOS uncoupling.

### Antioxidant NAC attenuates myocardial I/R injury and post-ischemic cardiac dysfunction in diabetic rats

**Fig. 5:**
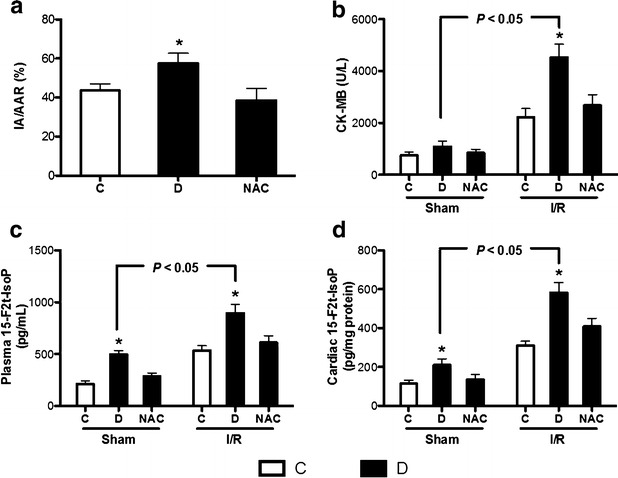
N-acetylcysteine attenuated myocardial ischemia–reperfusion (I/R) injury in diabetic rats. Control (*C*) or STZ-induced diabetic rats were either untreated (*D*) or treated with antioxidant N-acetylcysteine (1.5 g/kg/day, NAC) by oral gavage for 4 weeks. Then the rats were anaesthetized and subjected to myocardial I/R achieved by occluding the left anterior descending coronary artery for 30 min followed by reperfusion for 2 h. Sham-operated rats underwent the same surgical procedures without ligation. a–d infarct size (**a**), CK-MB (**b**), plasma 15-F2t-Isoprostane (15-F2t-IsoP, **c**), and cardiac 15-F2t-IsoP (**d**). All the results are expressed as mean ± SD, n = 7. Differences of infarct size among the groups were determined by using one-way ANOVA followed by Tukey’s test, the others were determined by using two-way repeated-measures ANOVA followed by Bonferroni’s post hoc test. *P < 0.05 vs. the other groups in sham or I/R conditions

We next investigate the treatment effects of NAC on myocardial I/R injury in diabetes. As shown in Fig. 5a, the rats subjected to myocardial I/R in untreated diabetic showed larger infarct size than that in non-diabetic control rats, which was attenuated by NAC treatment. We also measured the biochemical markers of myocardial I/R injury and oxidative stress in the experiment rats. Compared with corresponding sham-operated rats, the levels of plasma CK-MB, plasma 15-F2t-IsoP and cardiac 15-F2t-IsoP were significantly increased in rats underwent myocardial I/R insult (Fig. 5b–d). Under baseline conditions, diabetes-induced increase of both plasma and cardiac 15-F2t-IsoP was significantly attenuated by NAC treatment, but no significant difference was shown in plasma CK-MB among control, untreated and NAC treated diabetic rats. Whereas in the rats underwent myocardial I/R insult, the untreated diabetic rats demonstrated significantly increased the levels of plasma CK-MB, plasma 15-F2t-IsoP and cardiac 15-F2t-IsoP as compared with control rats, and all these changes were attenuated by NAC treatment. We also monitored hemodynamics in experimental rats. As shown in Table 2, the levels of HR, LVSP, +dp/dt and −dp/dt in diabetic rats at baseline were significantly reduced as compared with control rats, and NAC could significantly increase +dp/dt and −dp/dt levels as compared with untreated diabetic rats. However, NAC treatment slightly but not significantly increased the levels of HR and LVSP in diabetic rats. After 2 h of reperfusion, the levels of HR, LVSP, +dp/dt and −dp/dt in all experimental rats were significantly decreased as compared with that at baseline, indicating cardiac dysfunction following myocardial I/R insult. The reduction of HR, LVSP, +dp/dt and −dp/dt in diabetic rats underwent 2 h of reperfusion was significantly attenuated by NAC treatment.

**Table 2:** Hemodynamics at baseline and 2 h of reperfusion in experimental rats

	Heart rate (bpm)	LVSP (mmHg)	+dp/dt (mmHg/s)	−dp/dt (mmHg/s)
*Baseline (10* *min before ischemia)*				
C	374 ± 14	122 ± 7	6735 ± 643	4839 ± 612
D	313 ± 12^#^	103 ± 5^#^	5121 ± 520^#^	3670 ± 507^#^
D + NAC	328 ± 15^#^	114 ± 6	6355 ± 437^$^	4635 ± 463^$^
*2* *h reperfusion*				
C	287 ± 11**	91 ± 5**	4658 ± 486*	3240 ± 447*
D	220 ± 13**^#^	64 ± 4**^#^	2476 ± 379*^#^	2134 ± 384*^#^
D + NAC	235 ± 15**^#^	87 ± 4**^$^	3980 ± 424*^$^	3102 ± 337*^$^

Control (*C*), diabetic (*D*) and N-acetylcysteine-treated diabetic rats (1.5 g/kg/day, D+NAC) were subjected to 30 min (min) of left anterior descending coronary artery occlusion followed by reperfusion for 2 h (h). The heart rate, left ventricular systolic pressure (LVSP), left ventricular maximum rate of increase of left ventricular developed pressure (+dp/dt) and maximum rate of decrease of left ventricular developed pressure (−dp/dt) were monitored at 10 min before ischemia (baseline) and 2 h after reperfusion. All the results are expressed as mean ± SD, n = 8. Differences in hemodynamics at baseline and 2 h of reperfusion were determined by using two-way repeated-measures ANOVA followed by Bonferroni’s post hoc test

* P < 0.05

** P < 0.01 vs. their corresponding baseline

^#^P < 0.05 vs. their corresponding C group

^$^P < 0.05 vs. their corresponding D group

### NAC attenuates HG and hypoxia/reoxygenation (H/R) induced cell injury, which requires intact Cav-3/eNOS signaling

**Fig. 6:**
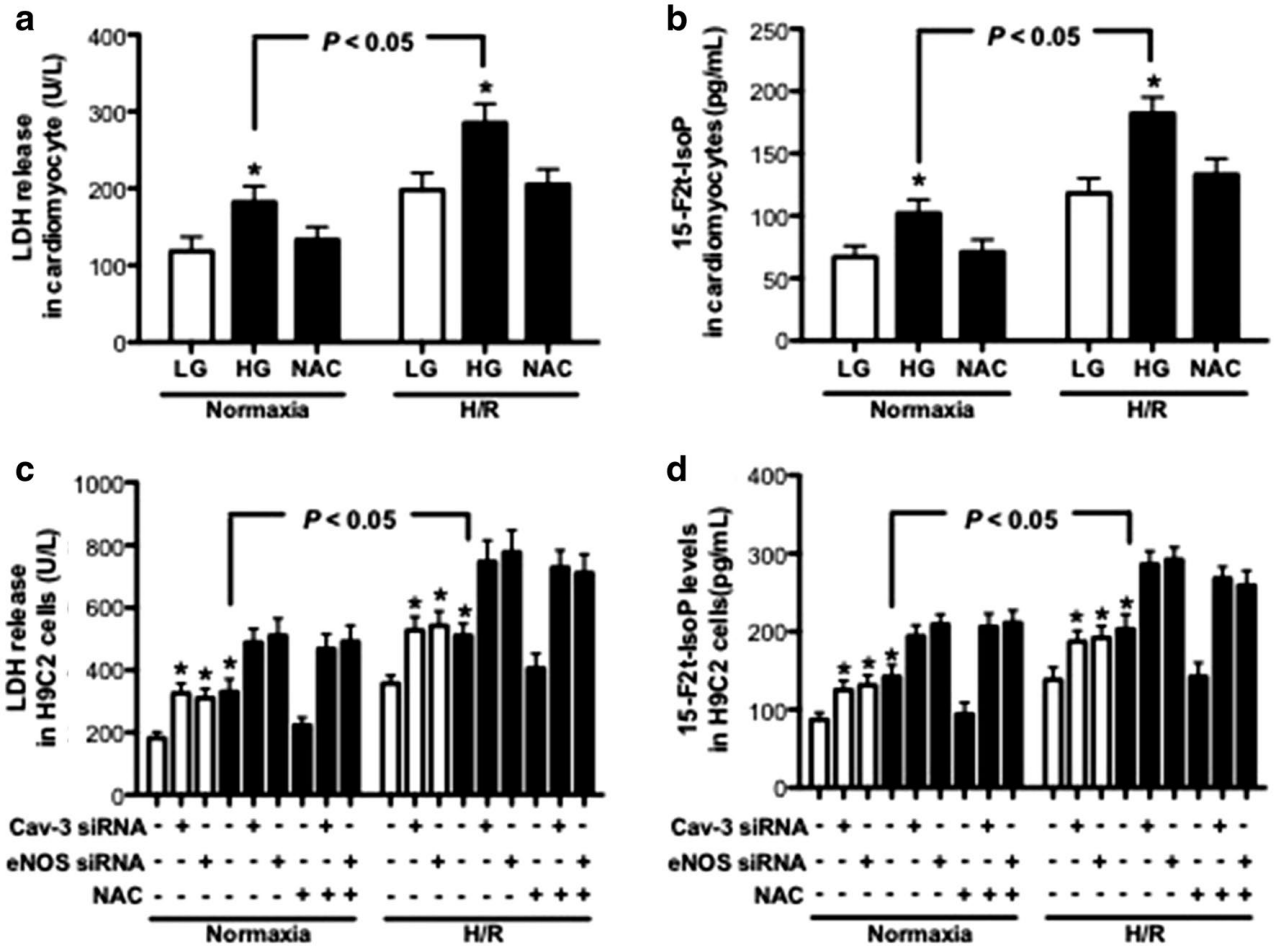
LDH release and 15-F2t-IsoP levels in cultured cardiomyocytes and H9C2 cells subjected to HG stimulation and hypoxia/reoxygenation (H/R) insult. Isolated cardiomyocytes from non-diabetic rats were treated with or without N-acetylcysteine (NAC, 1 mmol/L) during LG (5.5 mmol/L) or HG (25 mmol/L) conditions for 36 h, or H9C2 cells were transfected with Cav-3 siRNA or eNOS siRNA and treated with or without NAC during LG and HG conditions, then exposed to 4 h of hypoxia followed by 4 h of reoxygenation. **a**–**d** LDH release (**a**) and 15-F2t-IsoP (**b**) in cultured medium of cardiomyocytes, and LDH release (**c**) and 15-F2t-IsoP (**d**) in cultured medium of H9C2 cells. All the results are expressed as mean ± SD, n = 7. Differences were determined by using two-way repeated-measures ANOVA followed by Bonferroni’s post hoc test. *P < 0.05 vs. the other groups in normoxia or H/R conditions

We further determined the treatment effects of NAC on cultured cardiomyocytes and H9C2 cells exposed to H/R insult during LG and HG conditions. NAC and mannitol had no effects on cell injury measured by LDH and 15-F2t-IsoP during basal conditions (data not shown). As shown in Fig. 6, both the levels of LDH release and 15-F2t-IsoP in H/R-treated cardiomyocytes and H9C2 cells in various groups were significantly higher than that in the corresponding groups under normoxia conditions. HG stimulation significantly increased the levels of LDH release and 15-F2t-IsoP as compared with that in the LG group in both normoxia and H/R conditions, which were significantly attenuated by NAC treatment. To further investigate the role of Cav-3/eNOS in the beneficial effects of NAC in in vitro studies, the H9C2 cells were transfected with Cav-3 siRNA or eNOS siRNA during LG or HG conditions and treated with or without H/R insult. Similar to the effects of Cav-3 siRNA transfection on preventing Cav-3 expression (Fig. 4b), eNOS siRNA inhibited both the expression of total eNOS and eNOS phosphorylation at ser^1177^ in LG and HG conditions (data not shown). As shown in Fig. 6c, d, either Cav-3 siRNA or eNOS siRNA significantly increased LDH release and 15-F2t-IsoP levels in LG or HG conditions in H9C2 cells subjected to normoxia or H/R. Additionally, the concomitant treatment with Cav-3 siRNA or eNOS siRNA reversed NAC-mediated reduction in HG-induced LDH release and 15-F2t-IsoP production, indicating the beneficial effects of NAC on decreasing post-hypoxic cell injury require intact Cav-3/eNOS signaling.

## Discussion

In the present study, we have demonstrated that hyperglycemia-induced oxidative stress is accompanied with reduced Cav-3 expression and eNOS activation in diabetic hearts, which is consistent with our previous findings [20]. The advancement of the current study is that hyperglycemia-induced inhibition of eNOS activation is associated with caveolae dysfunction and reduced Cav-3 expression. Treatment with antioxidant NAC for 4 weeks attenuates diabetic cardiomyopathy and reduces myocardial I/R injury, possibly through improving Cav-3/eNOS/NO signaling. Furthermore, NAC treatment decreases 15-F2t-IsoP, O_2_^−^ and nitrotyrosine formation. The L-NAME-inhibited accumulation of O_2_^−^ suggests the presence of eNOS or NOS uncoupling, which is consistent with reduced cardiac levels of NO, p-eNOS (Ser^1177^). To our knowledge, this is the first study to explore the relationship between Cav-3 and eNOS in cardiomyocytes exposed to hyperglycemia and the effectiveness of antioxidant treatment.

### Treatment effects of NAC on diabetic cardiomyopathy and myocardial I/R injury

NAC is a thiol-containing radical scavenger and glutathione precursor, thus it is widely used as an antioxidant to remove ROS. Several studies have demonstrated that NAC may attenuate the myocardial damage by protecting cardiomyocytes from cell death [39], and NAC also has been proven to be effective in attenuating diabetic cardiac complications [22–28]. NAC when given at 0.5 g/kg/day for 3 months could partially attenuate hyperglycemia-induced myocardial oxidative stress and cardiac cell death in diabetic rats [40]. In comparison, NAC given at 1 g/kg/day, when treatment was started at an earlier phase and for a longer duration may be more effective in reducing ROS generation and fibrosis and improving cardiac function in diabetic mice [41]. In addition, NAC when administrated at the dose of 1.5 g/kg/day for 4 weeks could attenuate diabetes-induced cardiac damage [22–24] and myocardial I/R injury [25, 26]. In our preliminary studies, we have treated the diabetic rats with low (0.5 g/kg/day), medium (1 g/kg/day) and high dose (1.5 g/kg/day) of NAC for 4 weeks (data not shown), but only the high dose of NAC could significantly attenuated diabetic cardiomyopathy and myocardial I/R injury, so the high dose of NAC was chosen for ensuring mechanistic exploration in the present study.

Early diabetic cardiomyopathy is characterized by left ventricular diastolic dysfunction, reduced contractility and prolonged diastole [35, 42]. In the present study, diabetes decreased the ratio of E/A and increased left ventricular IVRT, but did not alter FS, FLVPW and EF as assessed by echocardiography, indicating myocardial diastolic function rather than systolic function is mainly impaired in the early STZ-induced diabetic rats, and this myocardial dysfunction was attenuated by antioxidant NAC treatment. It has been shown that NAC could decrease ROS levels in the fetal hearts and prevent the development of congenital heart disease in the offspring of pregestational diabetes [43] and attenuate the development of cardiac fibrosis and remodeling in a mouse model of heart failure [44]. Our present study showed that NAC treatment attenuated diabetes-induced myocardial hypertrophy evidenced by decreased ratio of heart weight to body weight and cardiomyocyte cross-sectional area. Similar to our study, chronic administration of sepiapterin and L-citrulline to increase eNOS activity preserves cardiac function and attenuates diabetic cardiomyopathy and myocardial I/R injury [45]. In the present study, the infarct size and CK-MB in untreated diabetic rats is higher than control rats, indicating diabetic rats are less tolerant to myocardial I/R injury, which is consistent with our previous study [29]. NAC treatment attenuated the reduction of HR, LVSP, +dp/dt and −dp/dt in diabetic rats underwent 2 h of post-ischemic reperfusion. Further, NAC treatment attenuated plasma and cardiac 15-F2t-IsoP (a specific marker of oxidative stress [30]) in diabetic rats both in sham and myocardial I/R conditions, and reduced infarct size and CK-MB levels in diabetic rats subjected to myocardial I/R insult.

### Role of hyperglycemia-induced oxidative stress in eNOS uncoupling

It is well documented that diabetes-induced cardiovascular diseases are associated with hyperglycemia-induced oxidative stress [2, 5, 6]. Excessive production of ROS may lead to impaired eNOS/NO signaling [7, 46], which is involved in diabetic cardiomyopathy and myocardial I/R injury in diabetic conditions [45, 47, 48]. In STZ-induced diabetic rats, we found increased cardiac 15-F2t-IsoP, nitrotyrosine and ROS production were concomitant with decreased eNOS phosphorylation at Ser^1177^ and NO levels. Our findings that diabetes induced increase of O_2_^−^ levels was blocked by the NOS inhibitor L-NAME in myocardial tissues and isolated cardiomyocytes, indicate the presence of eNOS and/or NOS uncoupling in diabetic heart, which is in line with a previous study [49]. Further studies showed that inhibition of Cav-3 expression by transfection with Cav-3 siRNA increased O_2_^−^ production, LDH release and 15-F2t-Isoprostane, and decreased eNOS activation and NO levels. This is in keeping with findings from a recent study showing that eNOS uncoupling is involved in caveolae dysfunction [50]. Furthermore, treatment of diabetes with antioxidant NAC reduced cardiac 15-F2t-IsoP, nitrotyrosine and O_2_^−^ production, and enhanced the protein expressions of Cav-3 and eNOS and their association, and increased eNOS phosphorylation and NO levels to levels comparable to that in the control rats. Thus, suppression of hyperglycemia-induced oxidative stress may restore eNOS activity and Cav-3 expression in diabetic hearts, and restoring the Cav-3 expression or increasing the association of eNOS and Cav-3 may attenuate eNOS uncoupling.

### Role of Cav-3/eNOS signaling in diabetic cardiomyopathy and myocardial I/R injury

Although the precise mechanisms by which hyperglycemia-induced inhibition of eNOS activity causes adverse effects in diabetic myocardium are not fully clear, the decreased expression of Cav-3, which is simultaneously stimulated by hyperglycemia in diabetes [19, 20], might be a major factor. The diabetes-induced decrease of Cav-3 expression may lead to caveolae dysfunction, which plays a vital role in affecting eNOS activity in diabetic hearts. This is well supported by our immunofluorescence studies demonstrating the colocalization of Cav-3 and eNOS in isolated cardiomyocytes during basal conditions. Immunoprecipitation experiments showed that diabetic conditions decreased the association of Cav-3 and eNOS in isolated cardiomyocytes, which was increased by NAC treatment. Caveolae disruption by methyl-β-cyclodextrin suppressed eNOS phosphorylation in isolated cardiomyocytes, and Cav-3 knockdown by siRNA prevented eNOS phosphorylation in H9C2 cells. These collectively suggest that caveolae or Cav-3 is required for eNOS activation in cardiomyocytes. NAC treatment attenuated HG-induced decrease of eNOS phosphorylation in isolated cardiomyocytes or H9C2 cells, but these effects of NAC were abolished in the presence of methyl-β-cyclodextrin and Cav-3 siRNA. This indicates that NAC restores eNOS activity possibly through improving Cav-3 expression or caveolae function. To our knowledge, this is the first study to provide direct evidence to demonstrate the relationship between decreased eNOS activity and caveolae dysfunction or reduced Cav-3 expression in cardiomyocytes and their link to hyperglycemia-induced oxidative stress.

Cav-3 is a major type of the caveolin family proteins in cardiac myocytes to form cardiomyocyte caveolae [17], which serve as a platform in the plasma membrane to modulate transduction pathways via signaling molecules docked within caveolins. It has been shown that both caveolae and caveolin-3 are critical for exendin-4 induced protection of the heart from I/R injury [51]. Heterozygous Cav-3 mice show increased susceptibility to palmitae-induced insulin resistance [52], and loss of Cav-3 expression results in diabetic cardiomyopathy [53]. Diabetes-induced decrease of Cav-3 expression may lead to the reduced tolerance to myocardial I/R injury [19, 21]. Additionally, Cav-3 overexpression attenuates cardiac hypertrophy [54] and induces endogenous cardiac protection from myocardial I/R injury [55]. This indicates that enhancing Cav-3 expression is beneficial to attenuate diabetic cardiomyopathy and myocardial I/R injury. In the present study, HG exposure for 36 h decreased Cav-3 expression in isolated cardiomyocytes or H9C2 cells, which were consistent with deceased Cav-3 in hearts from 5 weeks duration of diabetes. NAC treatment-induced restoration of Cav-3 expression attenuated diabetic cardiomyopathy and myocardial I/R injury in in vivo studies, and reduced HG and H/R induced cell injury in in vitro studies. However, the beneficial effects of NAC in H9C2 cells were abolished during concomitant treatment with Cav-3 siRNA or eNOS siRNA, suggesting the beneficial effects of NAC on diabetic hearts are achieved through Cav-3/eNOS signaling. These findings may have clinical implications in developing therapies to incorporate alternative antioxidant treatment and improving Cav-3/eNOS/NO signaling in combating diabetic cardiomyopathy and myocardial I/R injury.

## Conclusions

In summary, the results of current study demonstrate that hyperglycemia-induced inhibition of eNOS activity is associated with caveolae dysfunction and reduced Cav-3 expression in diabetic hearts. Antioxidant NAC attenuates myocardial dysfunction and myocardial I/R injury by improving Cav-3/eNOS/NO signaling. Our data suggest that antioxidant treatment and improving Cav-3/eNOS signaling may be useful approaches for correcting diabetes-induced abnormalities. However, further studies will be needed to investigate the underlying mechanisms using cardiac specific Cav-3 overexpression or Cav-3 knockout mice.

## Abbreviations

AAR: the area at risk
Cav-3: caveolin-3
CK-MB: creatine kinase-MB
DHE: dihydroethidium
+dp/dt: maximum rate of increase of left ventricular developed pressure
−dp/dt: maximum rate of decrease of left ventricular developed pressure
EF: ejection fraction
eNOS: endothelial nitric oxide synthase
FS: fractional shorting
H-E: haematoxylin and eosin
HG: high glucose
H/R: hypoxia/reoxygenation
I/R: ischemia–reperfusion
IVRT: isovolumic relaxation time
LDH: lactate dehydrogenase
LG: low glucose
LV: left ventricular
LVIDd: left ventricular internal dimensions at end diastole
LVIDs: left ventricular internal dimensions at end systole
LVPWd: left ventricular posterior wall dimensions at end diastole
LVPWs: left ventricular posterior wall dimensions at end systole
LVSP: left ventricular systolic pressure
LVVd: left ventricular end-distolic volume
LVVs: left ventricular end-systolic volume
NAC: N-acetylcysteine
NO: nitric oxide
NOS: nitric oxide synthase
ROS: reactive oxygen species
SV: stroke volume
TTC: triphenyltetrazolium chloride
15-F2t-IsoP: 15-F2t-isoprostane
